# Aberrant expression of thyroidal hormone receptor α exasperating mitochondrial dysfunction induced sarcopenia in aged mice

**DOI:** 10.18632/aging.205748

**Published:** 2024-04-18

**Authors:** Yunlu Sheng, Xiaoxia Zhu, Lijun Wei, Yuxin Zou, Xinyu Qi, Runqing Shi, Wenli Xu, Xiaodong Wang, Guoxian Ding, Yu Duan

**Affiliations:** 1 Division of Geriatric Endocrinology, The First Affiliated Hospital of Nanjing Medical University, Nanjing 210029, People’s Republic of China

**Keywords:** thyroid hormone receptor α, mitochondrial dysfunction, mitophagy, skeletal muscles, aging mice

## Abstract

Disrupted mitochondrial dynamics and mitophagy contribute to functional deterioration of skeletal muscle (SM) during aging, but the regulatory mechanisms are poorly understood. Our previous study demonstrated that the expression of thyroid hormone receptor α (TRα) decreased significantly in aged mice, suggesting that the alteration of thyroidal elements, especially the decreased TRα, might attenuate local THs action thus to cause the degeneration of SM with aging, while the underlying mechanism remains to be further explored. In this study, decreased expression of myogenic regulators Myf5, MyoD1, mitophagy markers Pink1, LC3II/I, p62, as well as mitochondrial dynamic factors Mfn1 and Opa1, accompanied by increased reactive oxygen species (ROS), showed concomitant changes with reduced TRα expression in aged mice. Further TRα loss- and gain-of-function studies in C2C12 revealed that silencing of TRα not only down-regulated the expression of above-mentioned myogenic regulators, mitophagy markers and mitochondrial dynamic factors, but also led to a significant decrease in mitochondrial activity and maximum respiratory capacity, as well as more mitochondrial ROS and damaged mitochondria. Notedly, overexpression of TRα could up-regulate the expression of those myogenic regulators, mitophagy markers and mitochondrial dynamic factors, meanwhile also led to an increase in mitochondrial activity and number. These results confirmed that TRα could concertedly regulate mitochondrial dynamics, autophagy, and activity, and myogenic regulators rhythmically altered with TRα expression. Summarily, these results suggested that the decline of TRα might cause the degeneration of SM with aging by regulating mitochondrial dynamics, mitophagy and myogenesis.

## INTRODUCTION

Thyroid hormones (THs) orchestrate developmental processes in the body during embryogenesis and post-natal life by modulating diversified gene expression. Skeletal muscle (SM) is an important THs-target tissue, and intrinsic homeostasis of THs determines proper operation of SM such as contractile function, bioenergetic metabolism, myogenesis and regeneration [1]. Based on clinical evidence, improper thyroidal hormone secretion, both hyperthyroidism and hypothyroidism, are associated with decreased skeletal muscle mass and muscle strength and related with sarcopenia [2, 3]. Actually, besides THs levels, the ultimate effects of THs on skeletal muscles mostly depend on tissue T3 bioavailability through several pivotal factors, including membrane transporters such as monocarboxylate transporter 8 (MCT8) and MCT10 sustained on myofibril; deiodinase 2 (DIO2) and DIO3 in myofibrillar cytoplasm which determine de iodinated activity of THs; and TH receptors α (TRα), the dominant receptor isoform in SM that regulates nuclear signal transduction of THs [1, 4]. The above-mentioned elements constitute the basis for cellular customization of TH signaling triad (TRIAD) in SM.

The muscle mass and muscle strength as well as muscle function would be attenuating in older adults, causing hazardous problems of health [5]. Our previous study in the euthyroid older adults showed that serum free triiodothyronine (FT3) level was positively related to muscle mass and muscle function [6]. This result suggests higher serum FT3 concentration within normal range maybe necessary and beneficial for sustaining the healthy status of SM during aging, while the underlying reason is unclear. In particular, the serum THs concentrations are quite stable and controlled by the Hypothalamic–Pituitary–Thyroid (HPT) axis, and systemic THs levels do not faithfully reflect their bioavailability in local tissues. Our subsequent study in different aged mice proved that instead of the variation of serum THs, the expression of TRα in SM decreased significantly with advancing age, suggesting that the alteration of thyroidal elements, prioritized of the decreased TRα in SM tissues, might attenuate local THs action thus to cause the degeneration of SM with aging [7]. However, the underlying mechanism remains unclear.

Mitochondria are critical organelles responsible for regulating the metabolic status of SM. The mitochondria supply the majority energy to cells through oxidative phosphorylation and maintain normal structure and performance of SM [8]. Reactive oxygen species (ROS), participated in mitochondrial dynamics, causing the organelles vulnerable to DNA mutations or protein misfolding [8]. To maintain mitochondrial homeostasis, mammalian cells have evolved several mitochondrial quality control systems such as mitochondrial dynamics, mitophagy and degradative pathways [9]. Mitochondrial dynamics are characterized by continuous fusion and fission. Mitochondrial fusion elongates the organelles, expands the mitochondrial network, and improves ATP synthesis and energy redistribution [10, 11]. The fusion between healthy and damaged mitochondria dilutes damaged material and avoids the accumulation of dysfunctional mitochondria in healthy networks [12], while the fission separates impaired components of mitochondria for further removal. Fusion and fission of the mitochondria are mediated by a variety of proteins such as Mfn1/2, Opa1 and Drp1. Mitophagy is a specific process that eliminates damaged organelle through autophagy to maintain the quality and quantity of the mitochondria. Mitochondrial dysfunction had intimate relationship with senescence and SM degeneration. TH signaling pathway has been proved to regulate mitochondrial quality control systems including mitochondrial dynamics and mitophagy, as well as mitochondrial function in SM [13–17]. However, the role of TRα in regulating mitochondrial quality control systems and mitochondrial function during aging is still unclear.

Together, our previous study revealed for the first time that the expression of TRα decreased significantly with advancing age in SM. Since thyroid hormone and its receptor, TRα determined more effect on mitochondrial quality control systems which were vital for keeping the health status of SM, and few studies revealed internal connection of TRα and mitochondria in SM aging, in this study, we detected the subtle changes of TRα and mitochondria in skeletal muscles in different aged mice, and further focused on the mitochondrial alterations caused by TRα loss- and gain-of-function “*in vitro*”, to explore the intrinsic link between TRα and mitochondrial function of SM.

We hypothesize that the decline of TRα might cause the degeneration of SM with aging by regulating mitochondria. Our study will lead to a better understanding of aging-associated sarcopenia and identify a novel therapeutic target.

## RESULTS

### Altered physical parameters of SM in mice during aging

**Figure 1:**
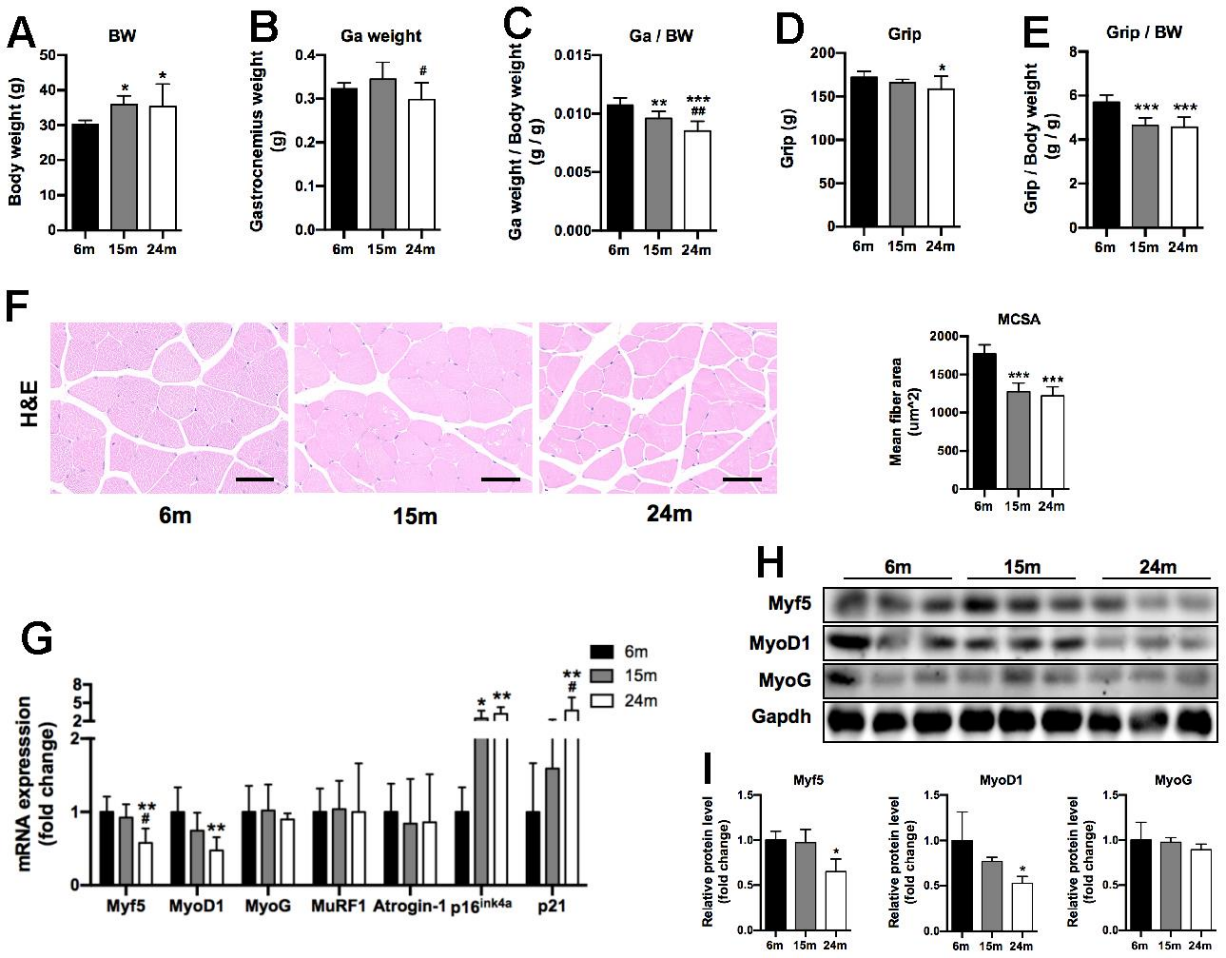
**Changes in body weight (BW), gastrocnemius muscle weight (Ga weight), four-limb grip strength (Grip), MCSA, and expression of myogenesis and senescence-related makers during aging.** (**A**) Body weight of mice; (**B**) Ga weight of mice; (**C**) Ga weight normalized by BW (Ga weight/BW); (**D**) Grip strength of mice; (**E**) Grip strength normalized by BW (Grip/BW), n=10 for 6m group, n=9 for 15m group, n=11 for 24m group (**A**–**E**); (**F**) Representative H&E staining images of Ga muscle section and quantitative analysis of MCSA level of three groups, Scale bar: 50 μm, n=3; (**G**) mRNA levels of myogenesis and senescence-related makers, n=7; (**H**) Representative Western blots and (**I**) quantification of Myf5, MyoD1, MyoG and Gapdh (loading control), n=3. *p<0.05, **p<0.01, ***p<0.001 vs. 6m group; #p<0.05, ##p<0.01 vs. 15m group.

Compared to 6m group, the body weight (BW) of 15m and 24m groups significantly increased (1.2-fold, p<0.05) (Figure 1A), while the Gastrocnemius (Ga) weight of 24m group significantly decreased by 14% compared to 15m group (p<0.05) (Figure 1B). Moreover, we observed age-related decline when Ga weight was corrected by BW (Ga/BW) (Figure 1C). Four-limb grip strength was attenuated in 24m group compared to 6m group (8% lower) (p<0.05) (Figure 1D). Consistently, the ratio of grip strength (Grip) to BW (Grip/BW) significantly reduced by about 20% in 15m and 24m groups compared to 6m group (p<0.001) (Figure 1E). Representative H&E staining images of SM section were shown in Figure 1F, and the myocyte cross sectional area (MCSA) was lower in 15m (28% lower) and 24m (30% lower) groups than in 6m group (P<0.001). As shown in Figure 1G, the mRNA levels of senescence-associated markers p16^ink4a^ and p21 increased with advancing age. The mRNA expression of MuRF1 and Atrogin-1 involved in muscle degradation showed no significant difference among three groups, while both mRNA (42% lower for Myf5, 53% lower for MyOD1) and protein (35% lower for Myf5, 47% lower for MyOD1) levels of myogenic regulatory factors Myf5 and MyoD1 significantly decreased in 24m group compared to 6m group (Figure 1G–1I).

### Increased ROS production and altered mitochondrial regulators of SM in mice with aging

**Figure 2:**
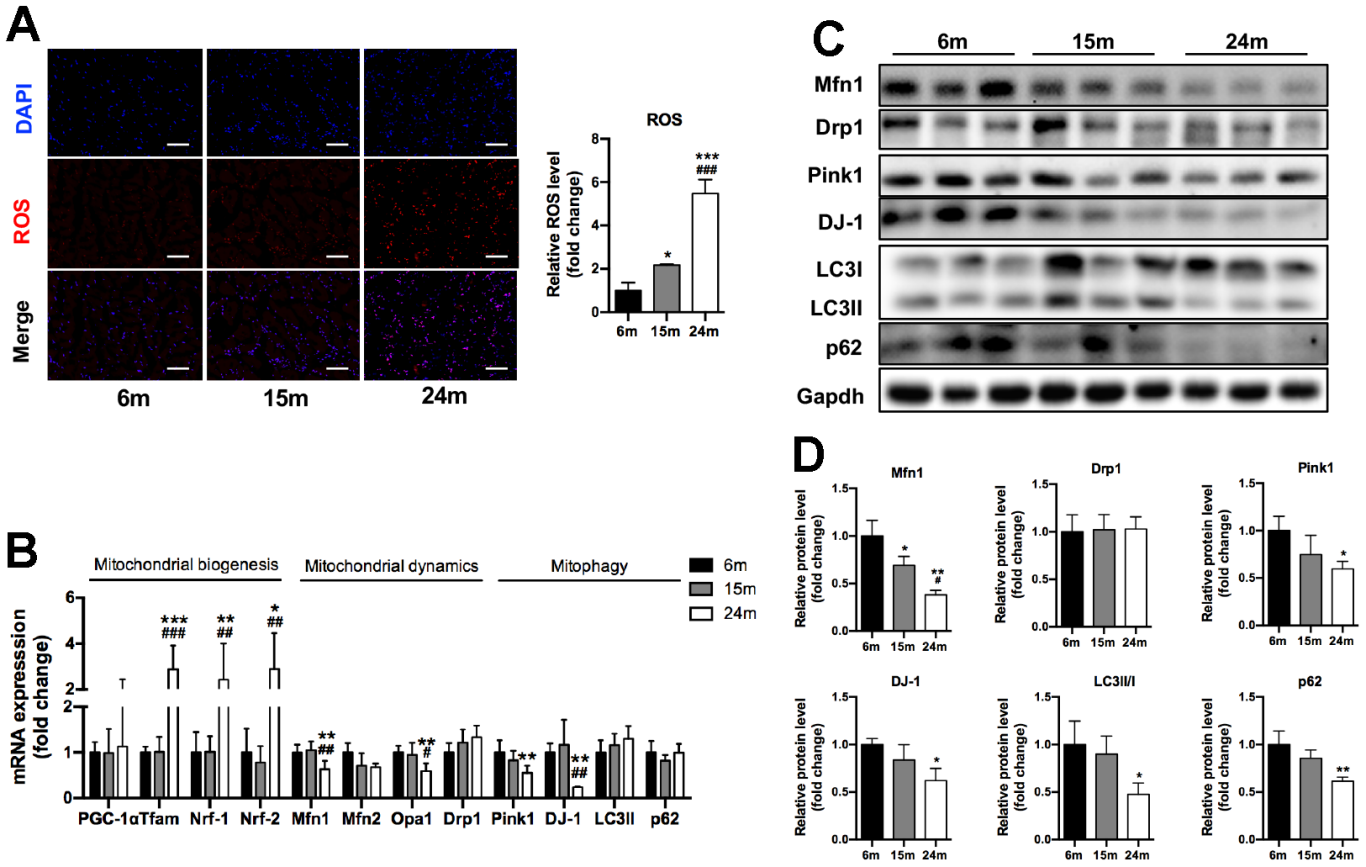
**Increased ROS production and altered mitochondrial regulators of SM in mice during aging.** (**A**) Representative ROS fluorescence staining of Ga muscles and quantification, Scale bar: 100 μm, n=3; (**B**) mRNA levels of mitochondrial biogenesis, mitochondrial dynamics and mitophagy/autophagy-related markers, n=7; (**C**) Representative Western blots and (**D**) quantification of Mfn1, Drp1, Pink1, DJ-1, LC3II/I, p62 and Gapdh (loading control), n=3. *p<0.05, **p<0.01, ***p<0.001 vs. 6m group; #p<0.05, ##p<0.01 vs. 15m group.

The high-energy SM is composed of a large number of mitochondria and the maintenance of mitochondrial integrity and function is critical for muscle health. Using immunofluorescence staining, we found that the ROS production (stained as red fluorescence) in SM significantly increased with aging (2.2-fold for 15m group, 5.5-fold for 24m group, both compared to 6m group, P<0.05) (Figure 2A). As shown in Figure 2B, the mRNA levels of mitochondrial biogenesis factors Tfam, Nrf-1, and Nrf-2 significantly increased in 24m group compared to 6m and 15m groups. Interestingly, the mRNA expression of mitochondrial dynamics factors Mfn1 (36% lower), Opa1 (40% lower) and mitophagy factors Pink1 (45% lower) and DJ-1 (22% lower) significantly decreased in 24m group compared to 6m group (p<0.01), while the mRNA expression of mitochondrial dynamics factors Mfn2 and Drp1, autophagy-related factors LC3 and p62 showed no difference among three groups (Figure 2B). As shown in Figure 2C, 2D, the protein expression of Mfn1 (62% lower), Pink1 (40% lower), DJ-1 (38% lower), LC3II/I (52% lower), p62 (39% lower) significantly decreased in 24m group, compared with 6m group (p<0.05), and Mfn1 protein expression significantly decreased by 31% in 15m group compared to 6m group (p<0.05).

In summary, these results demonstrated that mitochondrial regulators which involved in mitochondrial quality control, including mitochondrial dynamics, autophagy, and ROS deteriorated with aging in SM.

### Decreased expression of TRα in SM with aging

**Figure 3:**
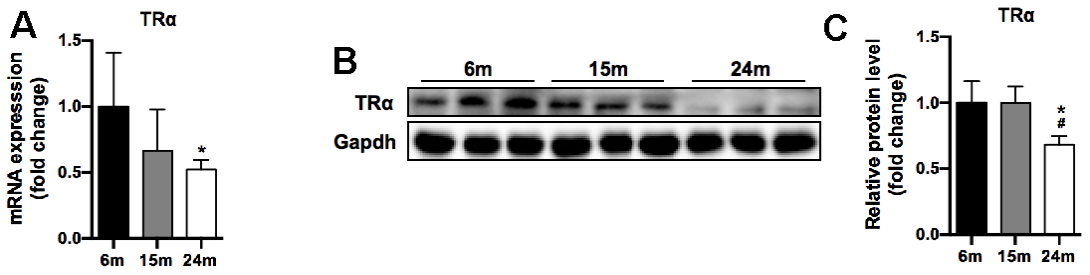
**TRα mRNA and protein levels of SM in mice during aging.** (**A**) mRNA level of TRα, n=7; (**B**) Representative Western blots and (**C**) quantification of TRα and Gapdh (loading control), n=3. *p<0.05 vs. 6m group; #p<0.05 vs. 15m group.

In our study, TRα mRNA level significantly decreased by 48% in 24m group compared to 6m group (p<0.05) (Figure 3A). Moreover, TRα protein level significantly decreased by 32% in 24m group compared to 6m and 15m groups (p<0.05), yet no significant difference between 6m and 15m groups (Figure 3B, 3C).

Considering the lower level of TRα observed in our study and its vital role in maintaining mitochondrial function according to previous studies [16, 17], we hypothesized that aging related muscle loss might result from the loss of TRα.

### The effects of TRα knockdown on mitochondrial dynamics, autophagy, function and myogenesis

**Figure 4:**
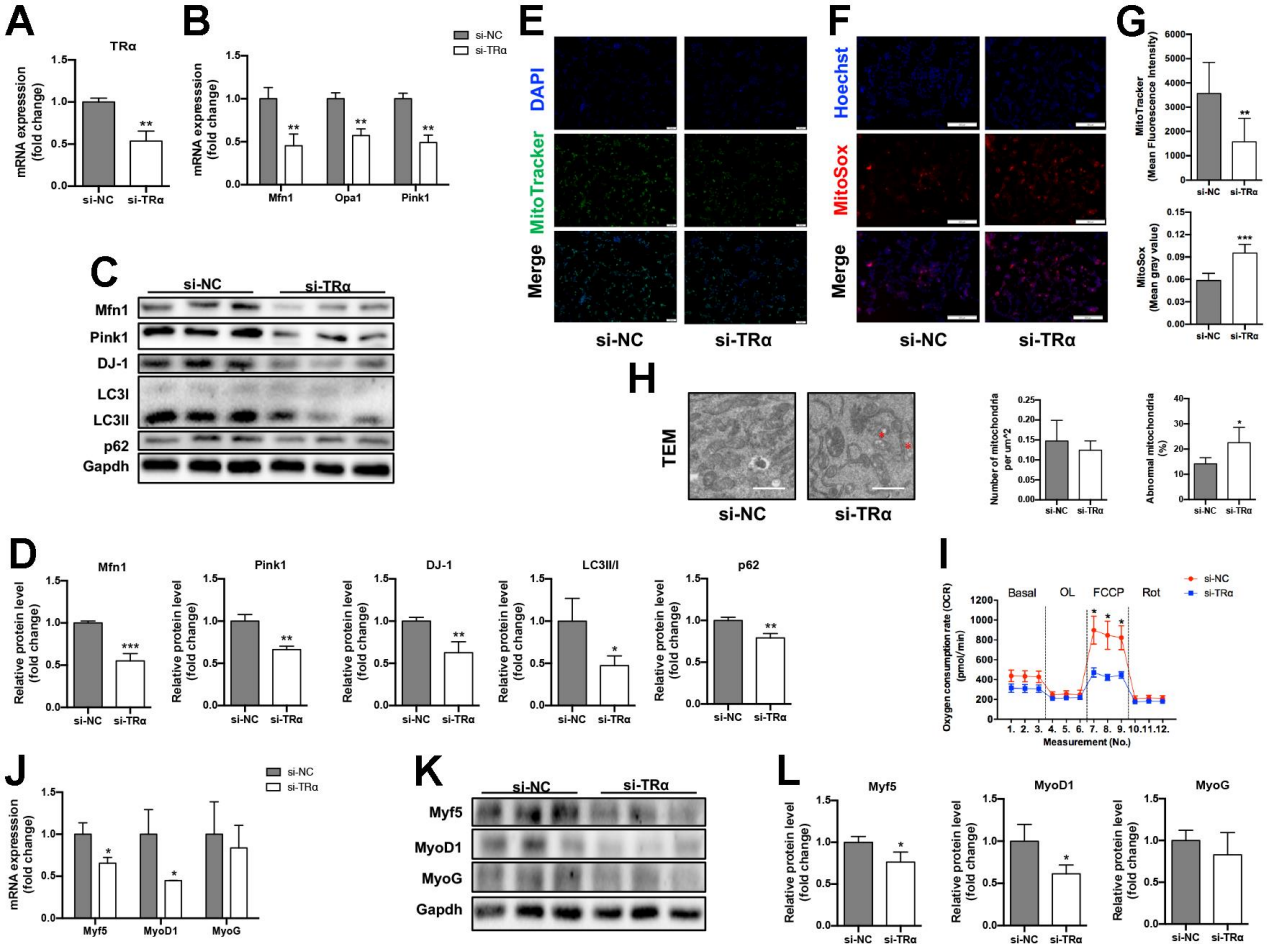
**Knockdown of TRα in C2C12.** The cells were transfected with TRα-targeted siRNA or non-targeted siRNA for 24h, 48h or 72h. si-NC negative control, si-TRα siRNA-TRα. (**A**) mRNA level of TRα, n=3; (**B**) mRNA levels of Mfn1, Opa1 and Pink1, n=3; (**C**) Representative Western blots and (**D**) quantification of Mfn1, Pink1, DJ-1, LC3II/I, p62 and Gapdh (loading control), n=3; (**E**) Representative MitoTracker immunofluorescence staining of cells, nuclei were counterstained with DAPI, scale bar: 100 μm; (**F**) Representative MitoSox immunofluorescence staining of cells, nuclei were counterstained with Hoechst, scale bar: 200 μm; (**G**) Quantification of MitoTracker Green fluorescence intensity (n=8) and MitoSox Red fluorescence intensity (n=5), respectively, fluorescence intensity was quantified using densitometric image analysis software with cell quantity adjustment; (**H**) Representative transmission electron microscopy images of mitochondria and quantification of mitochondrial number and percentage of abnormal mitochondria, the red ‘*’ represents damaged mitochondria (mitochondria with disrupted membrane, loss of cristae, matrix dissolution and vacuolization), scale bar: 1 μm, n=5; (**I**) Seahorse analysis of oxygen consumption rate (OCR), OCR was measured continuously throughout the experimental period at baseline and in the presence of the indicated drugs: 1.5 μM oligomycin, 0.5 μM FCCP and 0.5 μM rotenone, n=4; (**J**) mRNA levels of Myf5, MyoD1 and MyoG, n=3; (**K**) Representative Western blots and (**L**) quantification of Myf5, MyoD1, MyoG and Gapdh (loading control), n=3. *p<0.05, **p<0.01 vs. control group (si-NC). All experiments were repeated three independent times.

To investigate whether TRα regulates mitochondrial dynamics, autophagy, function or myogenesis, TRα-targeted siRNA (si-TRα) was constructed and transfected into C2C12 cells, with non-targeted siRNA (si-NC) as a control. si-TRα transfection successfully decreased TRα expression by 47% (P<0.01) (Figure 4A). Meanwhile, the mRNA expression of Mfn1 (55% lower), Opa1 (43% lower), and Pink1 (51% lower) significantly decreased (p<0.01) in si-TRα group compared to the control (Figure 4B). Consistently, the protein levels of Mfn1 (45% lower), Pink1 (34% lower), DJ-1 (38% lower), LC3II/I (53% lower), and p62 (21% lower) were significantly down-regulated (Figure 4C, 4D). Interestingly, silencing of TRα also led to a significant decrease (56% lower) in mitochondrial activity measured by MitoTracker immunofluorescence staining, higher (1.6-fold) mitochondrial ROS assessed by MitoSox immunofluorescence staining, and higher (1.6-fold) percent of damaged mitochondria, while no significant difference was observed in mitochondrial number measured by electron microscopy (Figure 4E–4H). Notedly, we also observed decreased (48% lower) oxygen consumption rate (OCR) stimulated by the mitochondrial uncoupler FCCP using a Seahorse Bioscience XF24 respirometry analyzer (Figure 4I). In addition, Myf5 and MyoD1 mRNA (34% lower for Myf5, 55% lower for MyOD1) and protein (24% lower for Myf5, 39% lower for MyOD1) expression significantly decreased in si-TRα group (p<0.05), while MyoG was slightly down-regulated in si-TRα group compared with the control group (Figure 4J–4L).

Together, these data indicated that loss of TRα expression in C2C12 might cause impaired mitochondrial dynamics, autophagy, function and myogenesis.

### The effects of TRα overexpression on mitochondrial dynamics, autophagy, function and myogenesis

**Figure 5:**
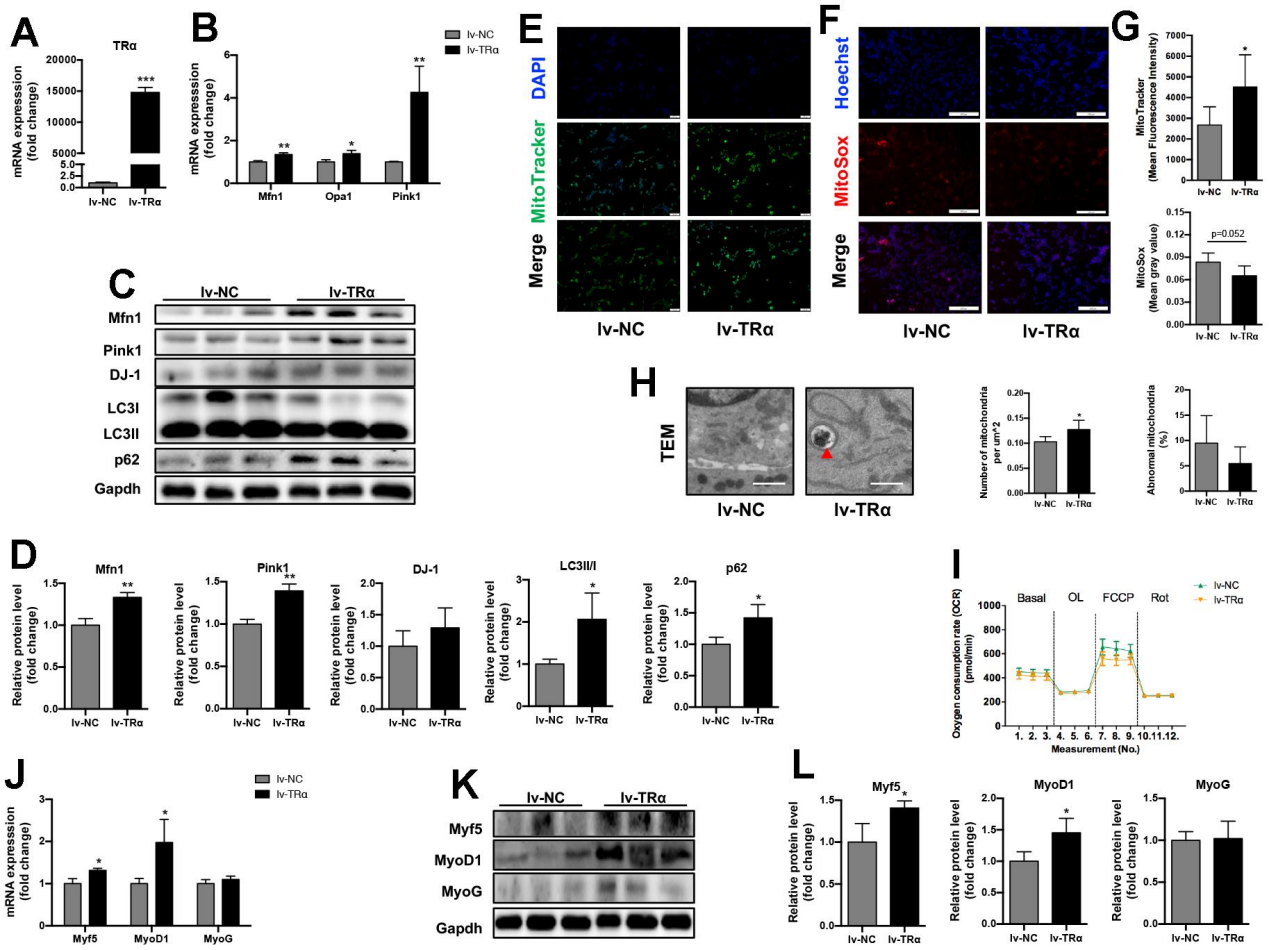
**Overexpression of TRα in C2C12.** Cells were transfected with TRα-targeted overexpression plasmid or non-targeted plasmid for 24h, 48h or 72h. lv-NC negative control, lv-TRα overexpressing plasmid-vector TRα. (**A**) mRNA level of TRα, n=3; (**B**) mRNA levels of Mfn1, Opa1 and Pink1, n=3; (**C**) Representative Western blots and (**D**) quantification of Mfn1, Pink1, DJ-1, LC3II/I, p62 and Gapdh (loading control), n=3; (**E**) Representative MitoTracker immunofluorescence staining of cells, nuclei were counterstained with DAPI, scale bar: 100 μm; (**F**) Representative MitoSox immunofluorescence staining of cells, nuclei were counterstained with Hoechst, scale bar: 200 μm; (**G**) Quantification of MitoTracker Green fluorescence intensity (n=8) and MitoSox Red fluorescence intensity (n=5), respectively, fluorescence intensity was quantified using densitometric image analysis software with cell quantity adjustment; (**H**) Representative transmission electron microscopy images of mitochondria and quantification of mitochondrial number and percentage of abnormal mitochondria, the red ‘▲’ is labeling mitochondria inside autophagic vesicles, Scale bar: 1 μm, n=5; (**I**) Seahorse analysis of oxygen consumption rate (OCR), OCR was measured continuously throughout the experimental period at baseline and in the presence of the indicated drugs: 1.5 μM oligomycin, 0.5 μM FCCP and 0.5 μM rotenone, n=4; (**J**) mRNA levels of Myf5, MyoD1 and MyoG, n=3; (**K**) Representative Western blots and (**L**) quantification of Myf5, MyoD1, MyoG and Gapdh (loading control), n=3. *p<0.05, **p<0.01 vs. control group (lv-NC). All experiments were repeated three independent times.

As shown in Figure 5B, accompanied by the increase of TRα expression (14779-fold, P<0.01) (Figure 5A), the mRNA expression of Mfn1 (1.3-fold), Opa1 (1.4-fold), and Pink1 (4.3-fold) significantly increased, and the protein expression of Mfn1 (1.3-fold), Pink1 (1.4-fold), LC3II/I (2.1-fold), and p62 (1.4-fold) also increased in lv-TRα group compared to the control (p<0.05), while there was only an upward trend for DJ-1 (Figure 5C, 5D). Notedly, overexpression of TRα also led to an increase in mitochondrial activity (1.7-fold) and number (1.2-fold, P<0.05), while there were only downward trends for mitochondrial ROS (22% lower, P=0.052) and percent of damaged mitochondria (43% lower, P=0.412) (Figure 5E–5H). No significant difference was observed in OCR (Figure 5I). Additionally, Myf5, MyoD1 mRNA (1.3-fold for Myf5, 2.0-fold for MyOD1) and protein (1.4-fold for Myf5, 1.5-fold for MyOD1) expression significantly increased when TRα was overexpressed (p<0.05), while MyoG expression showed no significant change (Figure 5J–5L).

Taken together, these results indicated that overexpression of TRα in C2C12 might improve mitochondrial dynamics, autophagy, activity and myogenesis.

## DISCUSSION

Thyroid hormone signaling plays a key role in maintaining functional homeostasis of SM, promoting myogenesis, accelerating growth and development of tissues, and controlling the metabolism of the substances in SM. Once the balance is broken, a series of myopathic changes may cause pseudohypertrophy, muscle weakness and muscle atrophy, indicating the crucial effects of THs on SM phenotypes and functions [1, 18]. The active form of THs, L-triiodothyronine (T3), regulates gene expression by binding its nuclear receptors. TRα and TRβ, two types of thyroid receptor, are heterogeneously sustained in disparate tissues, and TRα is predominantly expressed in brain, heart, and SM.

It is well known that the gradual loss of muscle mass and strength with age is the key factor leading to the decline of physical function in the older adults [5]. In this study we demonstrated that in aged mice, accompanying with decreased muscle weight and limb grip strength, senescent symbolic genes p16^ink4a^ and p21 were up-regulated while myogenic regulatory factors Myf5, MyoD1 were down-regulated, indicating that muscle content and strength, as well as myogenic ability consistently decrease with aging, which are the characteristics of senility in aged mice [19].

In the study, we found the decreased expression of TRα in both mRNA and protein levels in 24m aged mice, and the expression of mitophagy factors Pink1, DJ-1, LC3II/I, p62 and mitochondrial dynamics proteins Mfn1, Opa1 also decreased with age. In addition, using loss- and gain-of-function approach to knockdown and overexpress TRα, we showed that TRα could regulate mitophagy, mitochondrial dynamics and myogenesis. In a recent study, Zhou et al*.* demonstrated that TRα abnormality would inhibit normal function of mitophagy, reducing mitochondrial biogenesis and impairing lipid catabolism in TRα-mutant mice [17]. These results suggest that TRα acts as a prominent factor in SM aging process through regulating mitochondrial function, such as mitochondrial dynamics, mitophagy, and myogenesis.

Mitophagy is important to keep healthy status of the mitochondria by removing spoiled or senescent mitochondria through autophagy. Impaired mitophagy or mitochondrial dynamics contributes to sarcopenia, which is characterized by progressive decline in SM mass, strength or athletic function most frequently occurring with aging [20]. Pink1 is one of the prominent factors involved in mitophagy, it normally accumulates on the outer mitochondrial membrane, promotes protein ubiquitination, recruits autophagy receptors like p62/SQSTM1, and attracts autophagy receptors binding ubiquitinated cargo and LC3-coated phagophores to mediate autophagy [21], thus eliminating damaged organelles, repairing membrane integrity and maintaining mitochondrial homeostasis. The up-regulation of p62 and Pink1 would prolong lifespan in middle-aged drosophila [22, 23]. Our results showed that Pink1, DJ-1, LC3II/I and p62 expression significantly decreased in aged mice, which may be responsible for the decline of SM performance. Meanwhile, we demonstrated that TRα could regulate the expression of Pink1, DJ-1, LC3II/I and p62, suggesting that TRα mediated the balance of mitophagy in SM with aging.

The mitochondria are highly dynamic organelles that are constantly undergoing fission and fusion to adapt their morphology to cellular environment. Mitochondrial fusion is mediated by Mfn1/2 on the outer mitochondrial membrane and by Opa1 on the inner mitochondrial membrane, resulting in mitochondrial elongation [20]. Mfn1 and Mfn2 have a high degree of homology. The lack of either Mfn1 or Mfn2 could lead to mitochondrial dysfunction and decreased exercise performance, while genetic loss of Mfn1 could lead to a higher degree of mitochondrial fragmentation compared to Mfn2 deletion [24, 25]. Specific deletion of Opa1 in SM impacted mitochondrial function, oxidative stress, systemic inflammation and muscle mass [26–28]. We detected that Mfn1 and Opa1 were significantly deficient in aged mice accompanied by the decrease of TRα, and the levels of Mfn1 and Opa1 would alter synchronously with the variation of TRα, thus we found the decrease of TRα in aging SM brought out the declining expression of Mfn1 and Opa1.

SM is primarily comprised of myotubes which bundle into myofibrils. Satellite cells (SCs), located in sarcolemma and endomysium of myotubes, is essential to muscle homeostasis and renewals. SCs could activate, mobilize and proliferate as myoblasts, which are regulated by MyoD1 and Myf5. Myogenic differentiation is activated when myoblast population is expanded. At the onset of differentiation, myoblasts express myogenin (MyoG) which allows them to fuse with other newly differentiated myoblasts or pre-existing myotubes and prevents the reversal of muscle cell differentiation [29, 30]. During myoblast differentiation, autophagy is required to regulate oxidative stress and mitochondria-mediated apoptotic signaling. Autophagy deficiency during myoblast differentiation results in higher cellular ROS generation, apoptosis and impaired myoblast fusion and differentiation [31]. The knockdown of Pink1 could lead to decreased mRNA levels of MyoG in differentiated C2C12 cells [32]. Our results showed that along with the significant decreased expression of autophagy factors, ROS production increased and MyoD1, Myf5, MyoG expression was down-regulated in SM of aged mice, suggesting that the myogenic function is impaired with aging. Furthermore, we found that the expression of myogenic regulatory factors Myf5 and MyoD1 in C2C12 cells varied with the alternation of TRα expression. The above results displayed that TRα to some extent, regulated the expression of myogenesis factors Myf5 and MyoD1, was related to the changes of Pink1, one of the important markers of mitophagy, yet these phenomena and its value need to be evaluated more.

In conclusion, our results indicated that TRα expression was significantly reduced with aging, and such decline of TRα might aggravate mitochondrial dysfunction and promoted degeneration of SM with aging.

## MATERIALS AND METHODS

### Animals

Thirty C57BL/6J male mice of different ages were obtained from SPF Biotechnology (Beijing, China) and maintained at 20-25° C with free access to water and food. Young (6 months old, 6m; n=10), middle-aged (15 months old, 15m; n=9), and old (24 months old, 24m; n=11) mice were included, body weight and the four-limb grip strength were recorded. After all animals were euthanized, gastrocnemius muscle tissues were excised from the lower limb and weighted, then fixed in 4% paraformaldehyde or stored at −80° C.

### Cell culture

C2C12 myoblasts were cultured in growth media (GM) consisting of high-glucose DMEM containing 10% fetal bovine serum (FBS) with 1% penicillin/streptomycin and incubated at 37°C, 5% CO_2_. Myoblast differentiation was induced by replacing GM with differentiation media (DM) consisting of DMEM supplemented with 2% horse serum and 1% penicillin/streptomycin. Cells were collected at 72 h (D3) following the addition of DM.

### Grip strength test

The four-limb grip strength was analyzed by a grip strength meter. The tested mouse was first allowed to grasp a grid with four paws, and then the tail of mouse was pulled backwards away from the grid until it released the grid. The pulling speed should be gentle and slow enough to let the mouse resist against the pulling force. The peak force was recorded once the mouse moved away from the grid. Each mouse was tested at least three times.

### Histology

Ga muscle tissues were fixed in 4% paraformaldehyde solution, embedded in paraffin, sectioned (8 μm thin), stained with hematoxylin-eosin (HE), and then observed under a microscopy. The myocyte cross sectional area was quantitated from six randomly selected images from three independent muscle samples in each group.

### ROS staining

The ROS levels in SM were detected by the dihydroethidium (DHE) staining (Bestbio, Shanghai, China). The images were observed by fluorescence microscopy (Olympus, Tokyo, Japan). Four pictures were taken for each sample, and three independent muscle samples were analyzed for each group.

### Small interfering RNA and overexpression plasmid transfection

The TRα-targeting siRNAs and overexpression plasmid are conducted and purchased from GenePharma (Shanghai, China). C2C12 cells were transfected with TRα siRNA/plasmid or non-targeting siRNA/plasmid (negative control, final siRNA concentration 20 μM) using Lipofectamine 3000 (Invitrogen, USA).

### Real-time PCR (RT-PCR)

Total RNA was extracted using RNAiso Plus (Takara, Shiga, Japan) and cDNA was synthesized with PrimeScript RT Master Mix Kit (Takara, Japan). RT-PCR was performed with TB-Green Kit (Takara, Japan) on StepOnePlus system (USA). The primer sequences are shown in Supplementary Table 1. Relative mRNA level was analyzed by 2^−ΔΔCT^ method.

### Western blotting

Total protein was isolated using the protein extraction kit (Beyotime, Shanghai, China). Western blot analysis was performed using standard protocol with primary antibodies for Gapdh, Mfn1, Myf5, MyoD1, MyoG (Cell Signaling Technology, USA), and TRα, Drp1, Pink1, DJ-1, LC3, p62 (Abcam, Cambridge, UK). Quantification of protein bands was performed with Image-J software (USA).

### Immunofluorescence staining

C2C12 cells (transfected with TRα-targeted siRNA/overexpression plasmid or non-targeting siRNA/plasmid for 24h) were incubated with PBS containing MitoTracker Green FM (200 nM/L, Thermo Fisher Scientific, USA, cod. M7514) or MitoSox Red Mitochondrial Superoxide Indicator (5 uM/L, Thermo Fisher Scientific, USA, cod. M36008) for 20 min at 37° C. 4’,6-Diamidino-2-phenylindole (DAPI, Cell Signaling Technology, USA) or Hoechst was used to counterstain the nuclei. The images were observed by fluorescence microscopy (Olympus, Tokyo, Japan). 5-8 pictures were taken from each group for further analysis. Fluorescence intensity was quantified using densitometric image analysis software with cell quantity adjustment.

### Transmission electron microscopy (TEM)

C2C12 cells (transfected with TRα-targeted siRNA/overexpression plasmid or non-targeting siRNA/plasmid for 24h) were fixed with 2.5% glutaraldehyde in sodium phosphate buffer and washed 3 times with phosphate buffered saline, then post-fixed with 1% osmium tetroxide and dehydrated with a series of alcohol with increasing concentration. After embedding samples in Araldite, ultra-thin sections were cut and double-stained with uranyl acetate and lead citrate. Next, ultrastructure of mitochondria and autophagic vesicles was observed using the Hitachi HT7800 transmission electron microscope (Tokyo, Japan). Mitochondria (total and abnormal) were quantified blindly in 5 images from each group.

### Measurement of O_2_ consumption

The oxygen consumption of C2C12 cells was measured on an XF24 respirometer (Seahorse Bioscience, USA) following the manufacturer's instructions. In brief, C2C12 cells (transfected with TRα-targeted siRNA/overexpression plasmid or non-targeting siRNA/plasmid for 24h) were cultured in 24-well XF cell culture microplates and differentiated as indicated (Seahorse Bioscience). Oxygen consumption rates (OCR) were measured at basal glucose levels, as well as with drugs disrupting the respiratory chain: oligomycin (ATP synthase inhibitor, 1.5 μM) (Agilent Technologies, USA) and FCCP (uncoupler, 0.5 μM) (Agilent Technologies). Finally, the mitochondrial respiration was blocked by 0.5 μM rotenone (Agilent Technologies). The residual OCR was considered non-mitochondrial respiration.

### Statistical analysis

Statistical analysis was performed using GraphPad Prism 8.0 software (USA). All results were represented as mean ± standard deviation. The variance of multiple groups was analyzed by one-way ANOVA, followed by Tukey post hoc analysis to specifically test individual differences between groups. And the unpaired Student’s t-test was used to compare two independent groups. P<0.05 indicated statistical significance.

## Supplementary Material

Supplementary Table 1
