# Supplementary material for: Aberrant expression of thyroidal hormone receptor α exasperating mitochondrial dysfunction induced sarcopenia in aged mice
Source: Aging (Albany NY). 2024 Apr 18;16(8):7141–52. doi: 10.18632/aging.205748 (PMC11087121; doi:10.18632/aging.205748)
Supplement: Supplementary Table 1 [file aging-16-205748-s001.pdf]

SUPPLEMENTARY TABLE

Supplementary Table 1. Sequences of primers used.

mRNAS		Primer sequence (5'-3')
TRα	Forward	GGTCACCAGATGGAAAGCGAA
	Reverse	CCTTGTCCCCACACACGA
Myf5	Forward	CCTGTCTGGTCCCGAAAGAAC
	Reverse	GACGTGATCCGATCCACAATG
MyoD1	Forward	CCACTCCGGGACATAGACTTG
	Reverse	AAAAGCGCAGGTCTGGTGAG
MyoG	Forward	GAGACATCCCCCTATTTCTACCA
	Reverse	GCTCAGTCCGCTCATAGCC
MuRF1	Forward	GACAGTCGCATTTCAAAGCA
	Reverse	AACGACCTCCAGACATGGAC
Atrogin-1	Forward	CTCTGTACCATGCCGTTCCT
	Reverse	GGCTGCTGAACAGATTCTCC
$p16^{ink4a}$	Forward	GCTCAACTACGGTGCAGATTC
	Reverse	GCACGATGTCTTGATGTCCC
p21	Forward	CGAGAACGGTGGAACTTTGAC
	Reverse	CCAGGGCTCAGGTAGACCTT
PGC-1α	Forward	AAGTGGTGTAGCGACCAATCG
	Reverse	AATGAGGGCAATCCGTCTTCA
Tfam	Forward	GAGCGTGCTAAAAGCACTGG
	Reverse	CCACAGGGCTGCAATTTTCC
Nrf-1	Forward	GGTGGGGGACAGATAGTCCT
	Reverse	GCTGTCCGATATCCTGGTGG
Nrf-2	Forward	CGCTGGAAAAAGAAGTGGGC
	Reverse	AGTGACTGACTGATGGCAGC
Mfn1	Forward	TGCCCTCTTGAGAGATGACC
	Reverse	AGAGCCGCTCATTCACCTTA
Mfn2	Forward	AGAACTGGACCCGGTTACCA
	Reverse	CACTTCGCTGATACCCCTGA
Opa1	Forward	TCAGCAAAGCTTACATGCAGA
	Reverse	TGCTTGGACTGGCTACATTTT
Drp1	Forward	ACTGATTCAATCCGTGATGAGT
	Reverse	GTAACCTATTCAGGGTCCTAGC
Pink1	Forward	CTCAAGTCCGACAACATCCT
	Reverse	GCCATTGCCACCACGCTCTA
DJ-1	Forward	AGCCGGGATCAAAGTCACTG
	Reverse	GGTCCCTGCGTTTTTGCATC
LC3II	Forward	AGCAGCATCCAACCAAAATC
	Reverse	CTGTGTCCGTTCACCAACAG
p62	Forward	GTGAATTCGCTCGCCGCTCGCTAT
	Reverse	CGTCTCGAGTGCCTGCTGACAACACCTA
β-actin	Forward	GACAACTTTGGCATCGTGGA
	Reverse	ATGCAGGGATGATGTTCTGG
